# A *HD‐ZIP III* gene, *PtrHB4,* is required for interfascicular cambium development in *Populus*

**DOI:** 10.1111/pbi.12830

**Published:** 2017-11-18

**Authors:** Yingying Zhu, Dongliang Song, Peng Xu, Jiayan Sun, Laigeng Li

**Affiliations:** ^1^ National Key Laboratory of Plant Molecular Genetics and CAS Center for Excellence in Molecular Plant Sciences Institute of Plant Physiology and Ecology Shanghai Institutes for Biological Sciences Chinese Academy of Sciences Shanghai China; ^2^ Present address: Warnell School of Forestry and Natural Resources and Department of Genetics University of Georgia Athens GA 30602 USA

**Keywords:** cambium activity, *HD‐ZIP III*, interfascicular cambium, secondary growth, *Populus*

## Abstract

Wood production is dependent on the activity of the vascular cambium, which develops from the fascicular and interfascicular cambia. However, little is known about the mechanisms controlling how the vascular cambium is developed in woody species. Here, we show that *PtrHB4*, belonging to the *Populus HD‐ZIP III* family, plays a critical role in the process of vascular cambium development. *PtrHB4* was specifically expressed in shoot tip and stem vascular tissue at an early developmental stage. Repression of *PtrHB4* caused defects in the development of the secondary vascular system due to failures in interfascicular cambium formation. By contrast, overexpression of *PtrHB4* induced cambium activity and xylem differentiation during secondary vascular development. Transcriptional analysis of *PtrHB4* repressed plants indicated that auxin response and cell proliferation were affected in the formation of the interfascicular cambium. Taken together, these results suggest that *PtrHB4* is required for interfascicular cambium formation to develop the vascular cambium in woody species.

## Introduction

The plant vascular system, which enabled green plants to successfully colonize terrestrial land, is not only a pipeline for the transport of water, nutrients, signalling molecules and other materials over long distances, but also a skeleton to provide mechanical support for vertical growth. The primary vascular system comprises of a group of discrete vascular bundles containing fascicular cambium, primary phloem and primary xylem. The primary vascular bundles are originated from procambium cells at the peripheral region of the rib zone of the shoot apical meristem (SAM). In perennial woody plants, fascicular cambium located at the centre of primary vascular bundles undergoes extension into the interfascicular region and generates interfascicular cambium tangentially to form a ring of vascular cambium. Then, the meristematic activity of the vascular cambium gives rise to the continuous production of cylindrical secondary vascular tissue (wood), which is a large source of sustainable energy and a sink for atmospheric carbon dioxide.

To date, molecular understanding regarding how the ring of vascular cambium is developed in woody species is limited due to challenges faced in performing forward genetic analysis in trees. The herbaceous species *Arabidopsis* has been used in several studies as a research model to screen for mutants or to induce secondary growth with hormone treatment (Chaffey *et al*., 2002; Davin *et al*., 2016; Ko *et al*., 2004; Zhang *et al*., 2011). A number of genes has been identified for their role in regulating *Arabidopsis* cambium activity (Agusti *et al*., 2011; Parker *et al*., 2003; Pineau *et al*., 2005; Suer *et al*., 2011). *WOX4*, a WUSCHEL‐related HOMEOBOX gene, regulated by CLE41/44 (CLAVATA3/ESR‐related 41/44)/TDIF (tracheary element differentiation inhibitory factor) peptide and its receptor PXY (PHLOEM INTERCALATED WITH XYLEM)/TDR (TDIF receptor), is required to promote cambial cells division (Baurle and Laux, 2005; Etchells and Turner, 2010; Hirakawa *et al*., 2008, 2010; Suer *et al*., 2011). *HCA2* (high cambial activity), a nuclear‐localized DNA binding with one finger (Dof) transcription factor *Dof5.6* promotes interfascicular cambium formation without alternating the organization of the vascular bundles in *Arabidopsis* inflorescence stems (Guo *et al*., 2009). MOL1 (MORE LATERAL GROWTH1) negatively regulates cambium activity by acting antagonistically to the CLE41/PXY/WOX4 cascade (Agusti *et al*., 2011; Gursanscky *et al*., 2016). Other receptor‐like kinases such as REDUCED IN LATERAL GROWTH1 (RUL1) act as an opposing regulator of cambium activity to MOL1 (Agusti *et al*., 2011). However, there are some developmental characteristics of secondary growth in woody species that may not be characterized using the *Arabidopsis* system. Secondary growth in *Arabidopsis* usually occurs at the basal part of inflorescence stems. The wall‐thickened interfascicular fibre cells, which are differentiated from interfascicular parenchyma cells, contribute to most of the basal secondary growth tissue. In contrast, in woody species, secondary growth originates from the meristematic activity of the vascular cambium, which forms vertically below the SAM via connecting the discrete fascicular/interfascicular cambia together (Larson, 1994; Nieminen *et al*., 2015; Philipson *et al*., 1971; Romberger *et al*., 1993; Sehr *et al*., 2010). Vascular cambium in woody plants produces secondary vascular tissue, which is precisely organized with vessel elements, fibre cells and ray parenchyma cells (Little *et al*., 2002; Mazur *et al*., 2014; Parker *et al*., 2003; Pineau *et al*., 2005). Thus, woody species are believed to have evolved specific molecular mechanisms to regulate the development of secondary growth such as controlling interfascicular cambium formation that have yet to be elucidated.

*HD‐ZIP III* gene family has been shown to act in both distinctive and redundant manners to regulate meristem function, organ polarity and vascular development (Emery *et al*., 2003; Izhaki and Bowman, 2007; McConnell *et al*., 2001; Prigge *et al*., 2005). *HD‐ZIP III* genes along with auxin, auxin polar transporter PINs and auxin response factor MP/ARF5, form an integrated feedback loop that is essential for the formation of the procambium during the development of the *Arabidopsis* embryo, leaf and root (Donner *et al*., 2009; Jouannet *et al*., 2015; Muller *et al*., 2016). During secondary growth in trees, auxin peaks are present in the cambium zone and developing xylem in a lateral gradient manner (Tuominen *et al*., 1997). Meanwhile, *Populus PIN* genes show the same expression pattern in the cambial zone (Schrader *et al*., 2003). *PoptrMP1*, a homologue of the *MP/ARF5* gene in *Populus,* is expressed specially in developing secondary xylem and its overexpression increases the expression of *HD‐ZIP III* genes (Johnson and Douglas, 2007). These data suggest that a conserved HD‐ZIP III‐auxin‐PIN‐MP/ARF5 signalling pathway may be shared between procambium formation and vascular cambium establishment.

In this study, a *HD‐ZIP III* gene *PtrHB4* (*Potri.001G372300*), which was observed to be highly expressed in vascular tissues in *Populus* (Zhu *et al*., 2013), was investigated for its role in the development of secondary growth in *Populus*. The results suggest *PtrHB4* is required for interfascicular cambium formation, likely via a mechanism which influences the process of auxin response during vascular cambium development in woody species.

## Results

### *PtrHB4* expression is correlated with the process of vascular cambium formation

**Figure 1:**
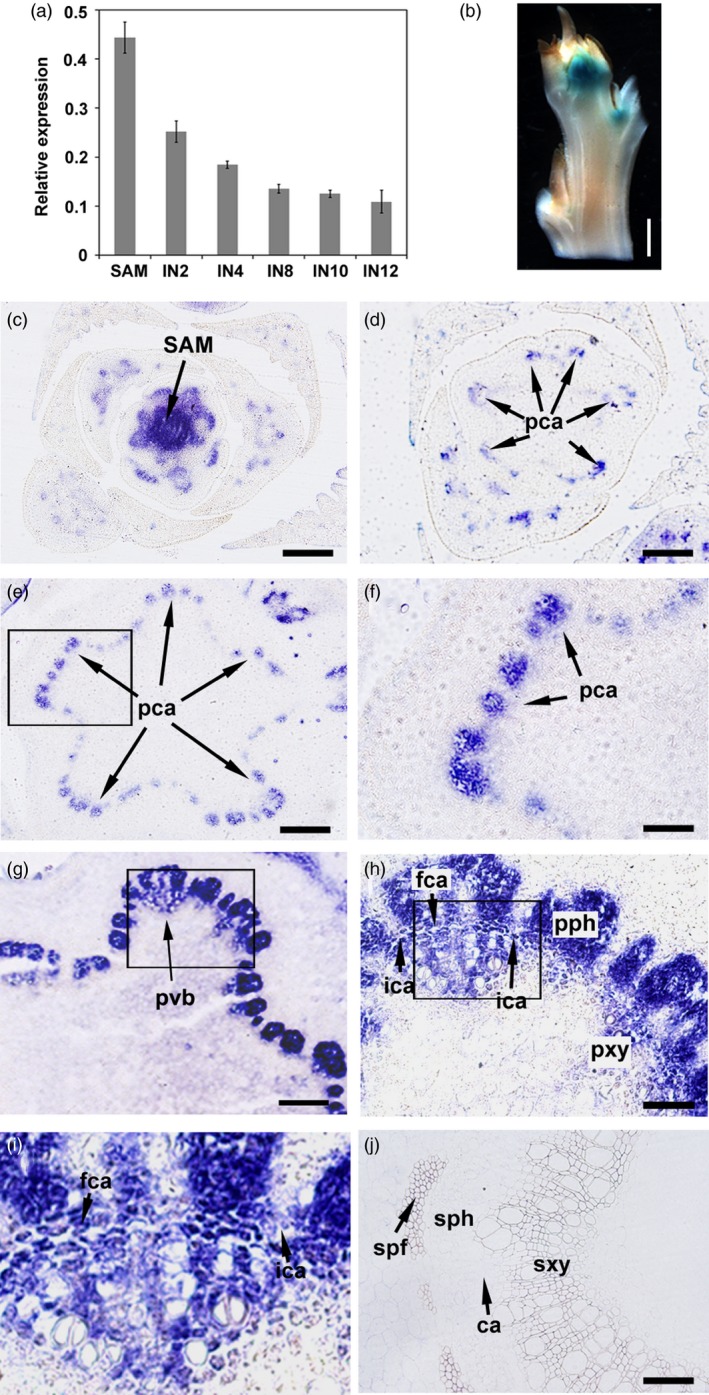
Expression pattern of *PtrHB4* during vascular cambium formation. (a) Expression of *PtrHB4* in *Populus* stem analysed by RT‐qPCR. *PtrActin1* was used as a reference gene. Bars are means ± SD of n = 3 biological replicates. (b) Histochemical analysis of GUS activity in shoot tip of *PtrHB4pro:GUS* transgenic plants. (c‐j) Immunolocalization analysis of PtrHB4 in shoot apex (c), in IN1 (d and e, indicating continual sections from top to bottom), (f) magnification of the framed section in (e). In IN2 (g), (h) magnification of the framed section in (g), (i) magnification of the framed section in (h) and in IN12 (j). IN, internode; SAM, shoot apical meristem; pca, procambium; ca, cambium; fca, fascicular cambium; ica, interfascicular cambium; ipc, interfascicular parenchyma cells; pvb, primary vascular bundle; pph, primary phloem; pxy, primary xylem; sph, secondary phloem; sxy, secondary xylem; spf, secondary phloem fibre. Bars: 1 mm in (b), 200 μm in (c), (d), (e) and (g), 100 μm in (f), (h) and (j), 20 μm in (i).

*PtrHB4* expression was examined from the top SAM tissue successively down to internodes (IN) undergoing secondary growth in the *Populus* stem. RT‐qPCR analysis indicated that expression of *PtrHB4* was prominent in shoot tip and young stem (IN2, IN4), but dramatically decreased in regions of the stem undergoing secondary growth (IN8, IN10 and IN12) (Figure 1a). A similar expression pattern was observed in *PtrHB4* promoter (*PtrHB4pro:GUS*) transgenic plants, in which strong GUS stain appeared in shoot tip as well as at the early development stage of vascular tissues (Figure 1b), while the GUS stain was barely detected in secondary vascular tissues (Figure S1a). To further analyse the location of PtrHB4 expression, PtrHB4‐specific antibodies were generated (Figure S1b‐e) and immunolocalization analyses were performed within the early development stage of vascular tissues. PtrHB4 was detected at the centre of tip including SAM with procambium cells and its surrounded region (Figure 1c and Figure S1f and g). As the vascular tissue expanded longitudinally, PtrHB4 protein was gradually restricted to primary vascular bundles in IN1 (Figure 1d–f, Figure S1f and g and Figure S2a and b). In IN2, PtrHB4 was localized in primary xylem, phloem and interfascicular parenchyma cells which can be induced to initiate cell division (Figure 1g–i and Figure S2e and f). Consistent with RT‐qPCR and the GUS assay, expression of the PtrHB4 protein was not found in the vascular tissues of IN12 undergoing secondary growth (Figure 1j). The expression pattern of PtrHB4 suggests it may play a role associated with the process of vascular cambium development.

### Repression of *PtrHB4* resulted in changes to secondary vascular tissue formation due to defects in interfascicular cambium development

**Figure 2:**
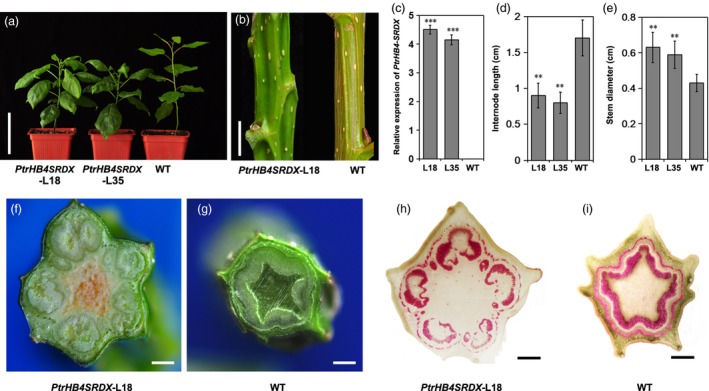
Repression of *PtrHB4* disrupted secondary vascular tissue development. (a) Morphological phenotype of *PtrHB4SRDX* (Line 18 and Line 35) and WT plants. (b) Stems of *PtrHB4SRDX* and WT plants. (c) Expression levels of *PtrHB4* in transgenic and WT plants. (d) Internode length and (e) stem diameter. Bars in (c), (d) and (e) are means ± SD of n = 3 biological replicates. Significance testing was conducted using the two‐sample *t*‐test (*P < 0.05, **P < 0.01 and ***P < 0.001). (f) and (g) Secondary vascular pattern in IN12 of *PtrHB4SRDX* and WT plants. (h) and (i) Cross sections of IN12 in *PtrHB4SRDX* and WT plants stained by phloroglucinol‐HCl. Bars: 10 cm in (a), 2 cm in (b), 5 mm in (f) and (g), 500 μm in (h) and (i).

To investigate the function of *PtrHB4* in *Populus*, a *PrtHB4* repressor was generated (*PtrHB4SRDX*) based on the chimeric repressor silencing technology (CRES‐T) (Figure S3) for circumventing the effects of *HD‐ZIP III* redundant genes (Hiratsu *et al*., 2003; Zhong *et al*., 2011). A total of thirty independent transgenic lines were generated and eighteen lines with increased *PtrHB4* expression (Figure 2c) showed similar phenotypes which were different from the wild type (WT) plant. *PtrHB4SRDX* plants displayed large, downward curling leaves (Figure 2a), although the number of leaves per *PtrHB4SRDX* plant was the same as WT plants in the same growth period (Figure 2a). *PtrHB4SRDX* plants grew shorter internodes and thicker stems compared to WT plants (Figure 2d and e). The stems of *PtrHB4SRDX* plants were polygonal prism shaped vs. WT plants which had cylindrical stems (Figure 2b). Cross sections of IN12 of *PtrHB4SRDX* plants showed that the vascular bundle‐like tissues were formed in an isolated manner, while WT plants had developed a cylindrical secondary vascular tissue (Figure 2f and g). Phloroglucinol‐HCl staining further indicated that each isolated vascular bundle exhibited closed xylem tissue while phloem tissue was formed surrounding the bundle structure (Figure 2h and i).

**Figure 3:**
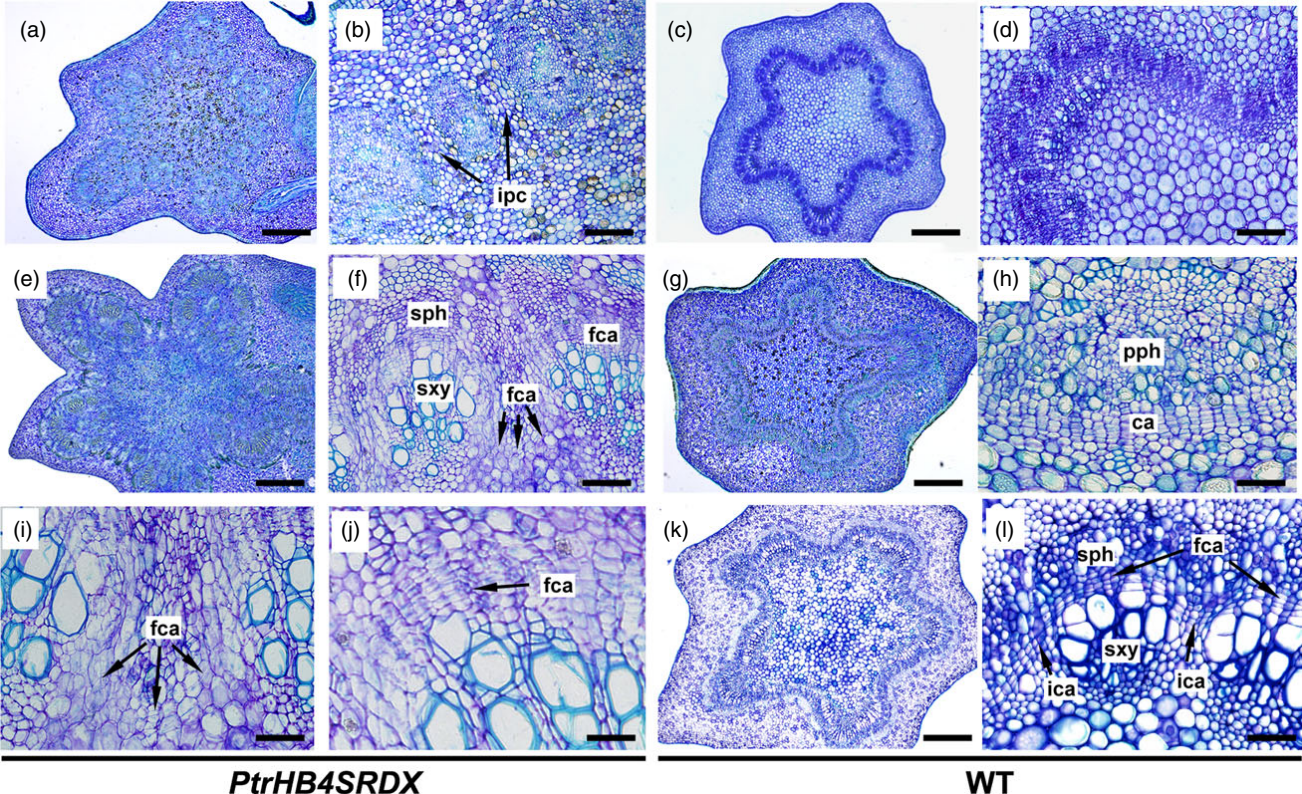
Repression of *PtrHB4* affected interfascicular cambium formation. (a) and (c) Cross section of the IN2 in *PtrHB4SRDX* and WT. (b) and (d) Magnification of (a) and (c). (e) and (k) Cross section of IN6 of *PtrHB4SRDX* and WT plants. (f) and (l) Magnification of (e) and (k). (g) Cross section of the IN3 in WT. (h) Magnification of (g). (i) Abnormal parenchyma cell division at the edge of fascicular cambium in *PtrHB4SRDX*. (j) Normal dividing activity of fascicular cambium in *PtrHB4SRDX* plants. ca, Cambium; fca, fascicular cambium; ipc, interfascicular parenchyma cells; sph, secondary phloem; sxy, secondary xylem. Bars: 500 μm in (a), (c), (e), (g) and (k), 100 μm in (b), (d), (f), 20 μm in (h), (i), (j) and (l).

*PtrHB4* was expressed as early as cambium initiation, and developmental defects were observed in the process of vascular cambium formation in *PtrHB4* repressed plants. Primary vascular bundles of both *PtrHB4SRDX* and WT plants were observed in IN2, but the distance between adjacent vascular bundles was larger in *PtrHB4SRDX* (Figure 3a and b) than in WT plants (Figure 3c and d). As early as IN3, the individual vascular bundles in WT plants had started to join each other through interfascicular cambium formation (Figure 3g and h). However, far to IN6 in *PtrHB4SRDX* plants, vascular bundles still failed to link together (Figure 3e and f), while at this stage, WT plants had started the formation of secondary vascular tissue (Figure 3k and l). Upon closer look, fascicular cambium cells appeared normal (Figure 3j), but the direction of cell division of parenchyma cells at the edge of fascicular cambium was changed in *PtrHB4SRDX* plants (Figure 3i). Consequently, the isolated vascular bundles developed into closed bundle tissues within IN16 of *PtrHB4SRDX* plants (Figure S4a, b, c and e). In addition, secondary xylem fibre cells were defective in terms of secondary cell wall formation and vessel elements showed smaller sizes with normal secondary cell wall deposition (Figure S4d and f). These results suggest that repression of *PtrHB4* affected cell division and led to failure in interfascicular cambium development.

### *PtrHB4* induced cambium activity and xylem cell differentiation

**Figure 4:**
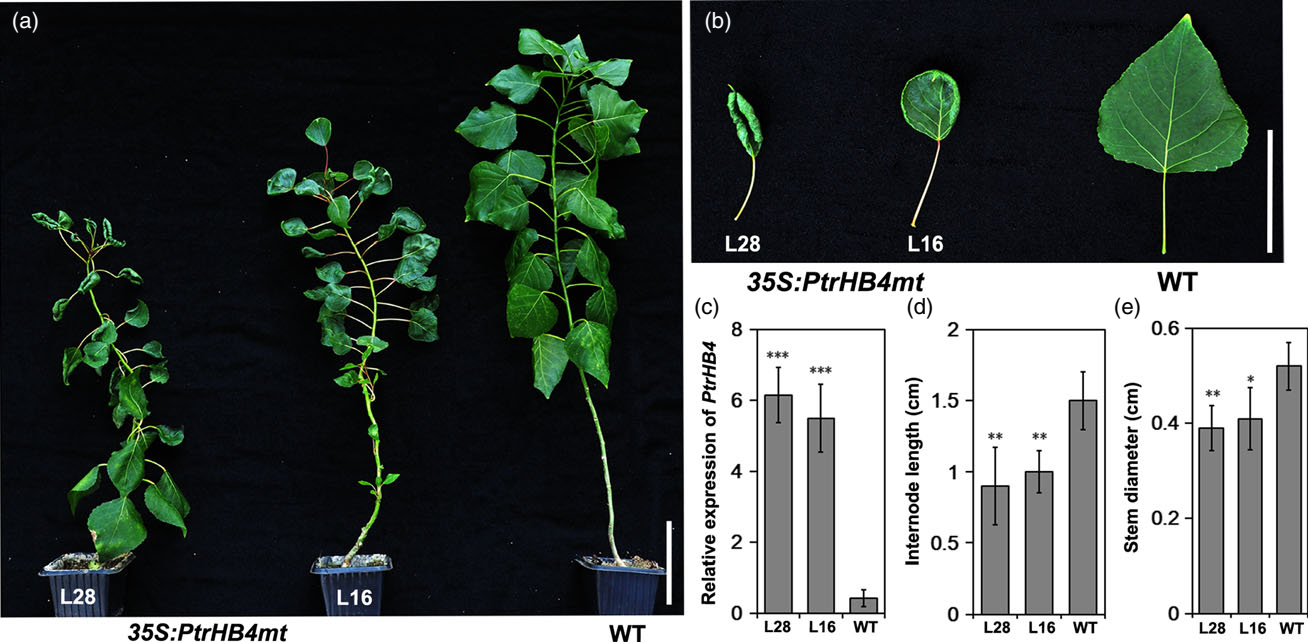
Morphological changes in overexpression of *PtrHB4mt Populus*. (a) Morphological phenotype of *PtrHB4mt* and WT plants. (b) Mature leaves of *PtrHB4*mt and WT plants. (c) Expression levels of *PtrHB4mt* in transgenic plants and WT plants. (d) Internode length and (e) stem diameter of *PtrHB4mt* and WT plants. Bars in (c), (d) and (e) are means ± SD of n = 3 biological replicates. Significance testing was conducted using the two‐sample *t‐*test (*P < 0.05, **P < 0.01 and ***P < 0.001). Bars: 10 cm in (a), 5 cm in (b).

**Figure 5:**
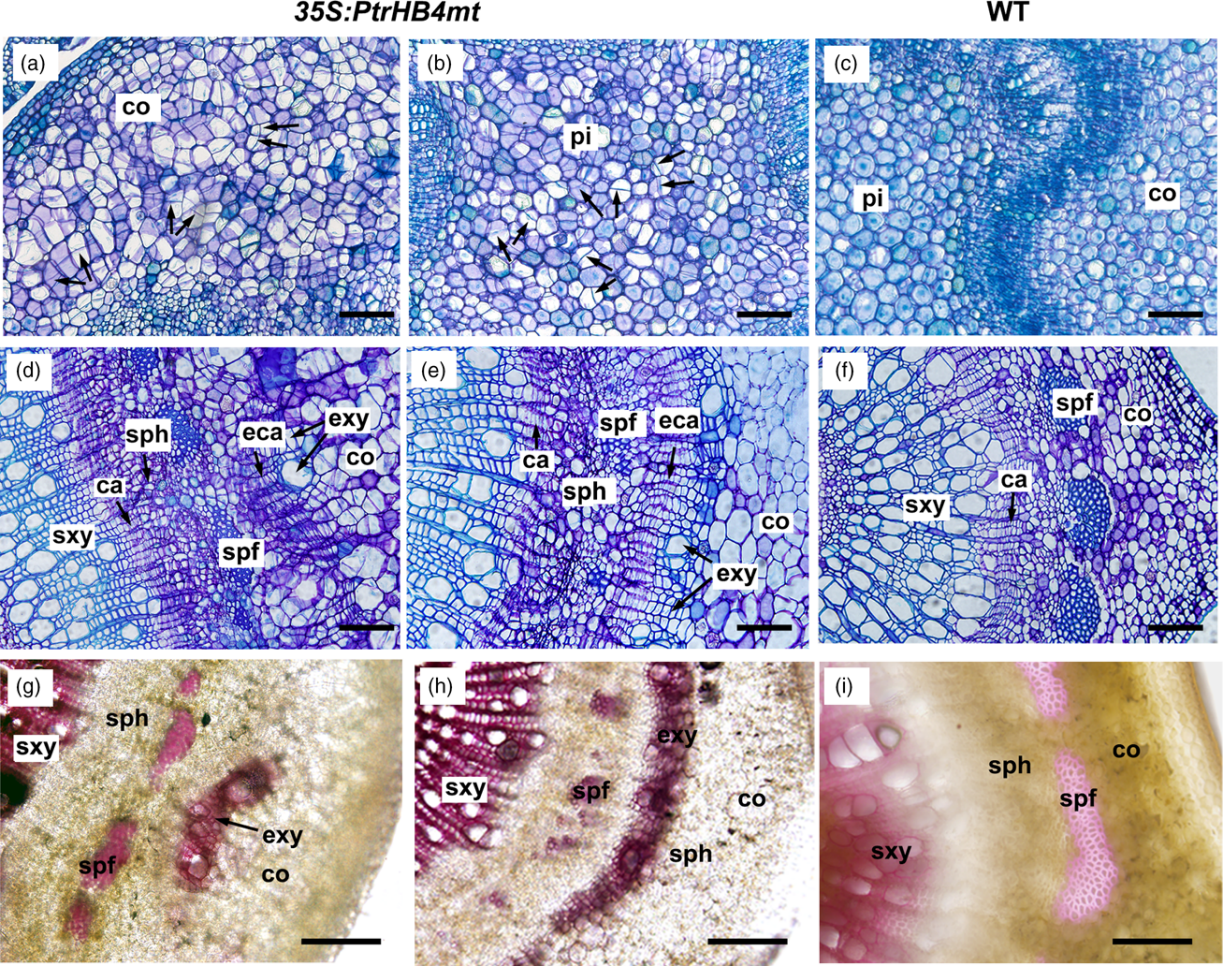
Overexpression of *PtrHB4mt* induced ectopic vascular cambium and xylem cells. Cross section of the IN2 in *PtrHB4mt* (a, cortex and b, pith) and WT plants (c). Cross section of the IN12 in *PtrHB4mt* (d and e, indicating different areas of cortex) and WT plants (f). Secondary cell wall staining by phloroglucinol‐HCl in the cortex of *PtrHB4mt* (g and h, indicating different areas of cortex) and WT plants (i). All sections were sampled from three‐month‐old plants. ca, Cambium; eca, ectopic cambium; sxy, secondary xylem; exy, ectopic xylem; sph, secondary phloem; spf, secondary phloem fibre; co, cortex. Bars: 20 μm in (a), (b) and (c), 100 μm in (d), (e), (f), (g), (h) and (i).

To obtain further evidence to aid in understanding of *PtrHB4* function, *PtrHB4* was mutated (*PtrHB4mt*) by replacing four nucleotides within the miR165/166 target sequence to avoid miRNA regulation. Then, the *PtrHB4mt* gene was transformed into *Populus* under the control of the CaMV35S promoter (Figure S5) (Mallory *et al*., 2004). Twenty‐six independent transgenic lines were identified with high expression of *PtrHB4mt* and similar phenotypic changes (Figure 4a and c). Mature leaves of *PtrHB4mt* plants were up‐curled and smaller than WT plants, while the number of leaves in each *PtrHB4mt* plant was similar with WT plants in the same growth period (Figure 4a and b). *PtrHB4mt* plants had thinner stems with twisted and shortened internodes (Figure 4a, d and e). Anatomical analysis showed that parenchyma cells, within the cortex and pith of IN2 in *PtrHB4mt* plants, displayed dividing activity, indicated by induction of cell division (Figure 5a, b and c). Within IN12, the vascular tissues appeared normal in *PtrHB4mt* plants (Figure S6a and b). Several layers of ectopic cambium cells were formed from parenchyma cells in cortex (Figure 5d and e). Further, the newly produced ectopic cambium cells were differentiated into xylem‐like cells (Figure 5d–f), which are morphologically similar to fibre cells and vessel elements as indicated by secondary cell wall staining (Figure 5g–i). Here, the results indicate that overexpression of *PtrHB4mt* led to induction of ectopic cambium and secondary xylem‐like cells in cortex parenchyma cells.

### *PtrHB4* may associate with the auxin signalling pathway during cambium formation

**Figure 6:**
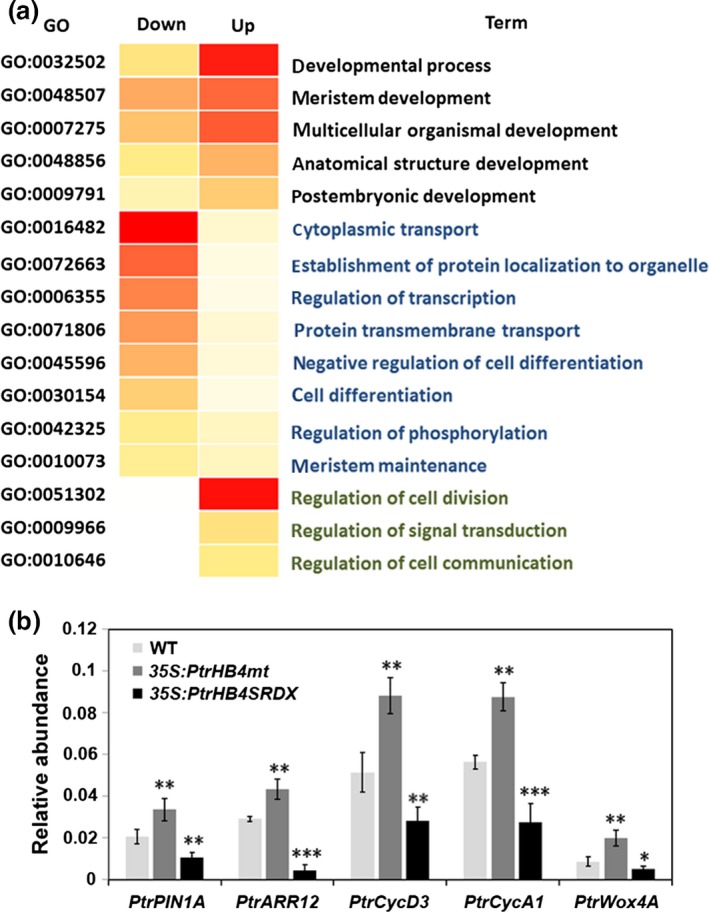
GO enrichment of differential expression genes and expression levels of genes related to *PtrHB4* signalling pathway. (a) Significantly overrepresented GO biological process terms for DE genes. (b) Expression analysis of *PtrPIN1A*,* PtrARR12*,* PtrCycD3*,* PtrCycA1*,* PtrWox4A* in *PtrHB4SRDX*,* PtrHB4mt* and WT plants. *PtrActin1* was used as a reference gene. Bars are means ± SD of n = 3 biological replicates of two independent lines of *PtrHB4SRDX* and *PtrHB4mt*. Significance testing was conducted using the two‐sample *t‐test* (*P ≦ 0.05, **P < 0.01 and ***P < 0.001).

To investigate how *PtrHB4* affects molecular processes during vascular cambium formation (Figure 1b), RNA sequencing of the shoot tip and IN1‐IN4 tissues from *PtrHB4SRDX* and WT plants was performed. Sequencing analysis showed that a total of 994 genes were differentially expressed (DE) due to *PtrHB4* repression (*PtrHB4SRDX* versus WT), among which 498 genes were down‐regulated and 496 genes were up‐regulated (5 ≤ log2 ratio (RPKM of *PtrHB4SRDX*/RPKM of WT) ≤−5 and *P* value ≤0.05) (Table S1 and S2). Gene ontology (GO) enrichment analysis indicated that the DE genes were associated with meristem development, postembryonic development and anatomical structure development. The GO categories for cell differentiation, protein localization and regulation of transcription were overrepresented among down‐regulated genes, while GO categories for cell division and regulation of signal transduction were enriched among up‐regulated genes (Figure 6a).

Genes related to auxin response were down‐regulated in *PtrHB4SRDX* plants, including *TIR1* (*Transport Inhibitor Response1*,* Potri.004G033900*), *AUX1* (*Auxin Resistant1*,* Potri.016G113600*) and *PIN1* (*PINFORMED1, Potri.015G038700*). Moreover, cyclin genes, cyclin‐dependent kinase genes and *ARR12* (*Potri.018G021300*) were down‐regulated in *PtrHB4SRDX* plants. In addition, the CLV/WUS signalling pathway‐related genes were down‐regulated in *PtrHB4SRDX* plants, including one ortholog of *CLV1* (*Potri.005G241500*), one ortholog of *WOX4* (*Potri.014G025300*), *POL* (*Poltergeist*,* Potri.002G185000*), *PLL4* (*Poltergeist like4*,* Potri.001G239300*) and *BRAD1* (*Breast Cancer Associated Ring1*,* Potri.002G259000*). An orthologue of *KANADI2* (*Potri.003G096300*) was up‐regulated in *PtrHB4SRDX* plants. Expression alternations of genes related to both auxin and cytokinin signalling pathways were further verified in both *PtrHB4SRDX* and *PtrHB4mt* plants by RT‐qPCR analysis (Figure 6b). Together, transcriptional analysis suggests that *PtrHB4* may associate with auxin and cytokinin signalling pathways, which in turn may be involved in *PtrHB4‐*mediated interfascicular formation processes during vascular cambium development.

## Discussion

Secondary growth in woody species is dependent on the meristematic activity of the vascular cambium. During the formation of the cylindrical vascular cambium, discrete primary vascular bundles need to connect to each other through interfascicular cambium development. Generally, interfascicular cambium is initiated by interfascicular parenchyma cells undergoing periclinal cell division adjacent to the fascicular cambium and then merged with the fascicular cambium to form a ring of vascular cambium (Eames and MacDaniels, 1947; Little *et al*., 2002; Mazur *et al*., 2014). However, little is known regarding how vascular cambium development is molecularly regulated. In this study, *PtrHB4*, a *HD‐ZIP III* gene in *Populus*, was showed to regulate interfascicular cambium formation in a developmental‐specific manner.

**Figure 7:**
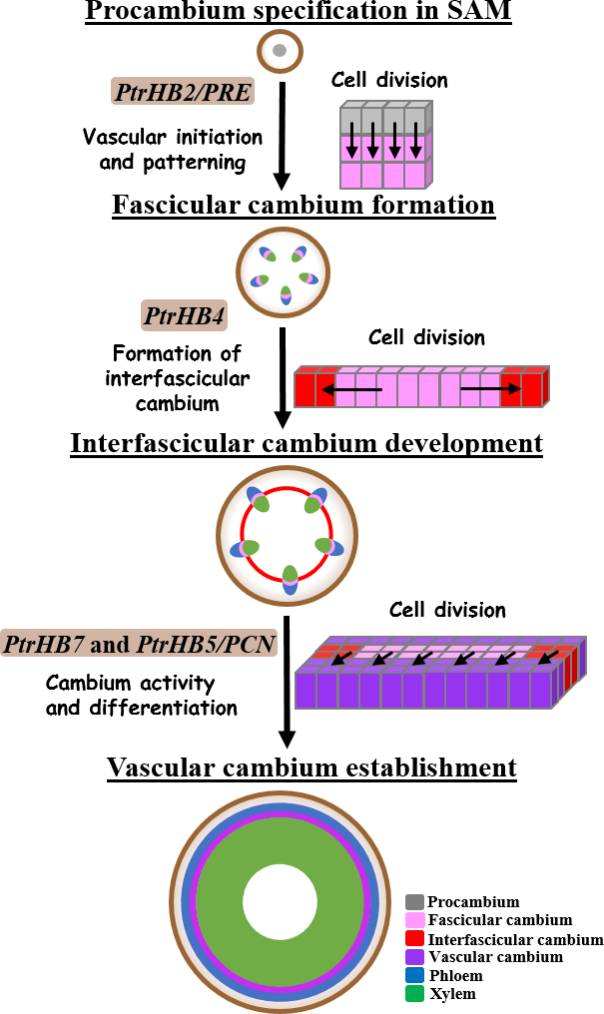
Functions of *HD‐ZIP III* genes during vascular development in *Populus*. A schematic view of distinctive roles of *HD‐ZIP III* genes in regulating vascular development process in the stem of *Populus*. Arrows represented the areas with cells undergoing vertical, periclinal or anticlinal division, characterized for following stages of cambium development.

To develop vascular cambium, the procambium in the rib zone right beneath SAM undergoes vertical division contributing to the initiation of the fascicular cambium in primary vascular bundles (Medford, 1992). As illustrated in Figure 7, interfascicular cambium is formed through periclinal division of parenchyma cells at the edge of fascicular cambium, which makes individual vascular bundles linking together to form a ring of vascular cambium system. Then, vascular cambium undergoes anticlinal division and differentiates secondary xylem and phloem (Larson, 1994; Little *et al*., 2002; Mazur *et al*., 2014; Philipson *et al*., 1971; Romberger *et al*., 1993). Repression of *PtrHB4* led to defective establishment of a ring of vascular cambium from fascicular and interfascicular cambia, likely due to the failure in initiation of interfascicular cambium, suggesting that *PtrHB4* was required for vascular cambium development. On the other hand, by overexpression of *PtrHB4* in *Populus*, normal ring of vascular cambium was retained, but ectopic cambium and xylem‐like cells were initiated from parenchyma cells in cortex, suggesting a *PtrHB4* role in induction of cambium activity. The results indicate that interfascicular cambium formation may be due to the capability of fascicular cambium activity regulated by *PtrHB4*.

*HD‐ZIPIII* gene family has indicated its ancestral role in vascular development and organ initiation (Prigge and Clark, 2006). It appears more specific function that *HD‐ZIP III* genes have displayed in the process of secondary growth in woody plants (Robischon *et al*., 2011; Zhu *et al*., 2013). Eight *HD‐ZIP III* genes (*PtrHB1* and *PtrHB2*,* PtrHB3* and *PtrHB4*,* PtrHB5* and *PtrHB6* and *PtrHB7* and *PtrHB8*) in *Populus* are divided into four clades and their distinctive expression patterns in *Populus* imply their function diversity in association with vascular development (Du *et al*., 2011; Zhu *et al*., 2013). As indicated in Figure 7, *PtrHB2/PRE* plays a major role in regulating initiation of cambium and patterning of vascular tissue (Medford, 1992; Robischon *et al*., 2011). *PtrHB7* promotes cambium activity and xylem differentiation (Zhu *et al*., 2013). *PtrHB5/PCN* inhibits cambium activity and xylem differentiation during secondary growth (Du *et al*., 2011; Zhu *et al*., 2013). Our evidence supports that *PtrHB4* plays a role in regulating the process of interfascicular cambium formation. On the other hand, overexpression of *PtrHB2/PRE*,* PtrHB4* and *PtrHB7* all induced ectopic cambium activity in cortex cells (Robischon *et al*., 2011), indicating that *PtrHB2/PRE*,* PtrHB4* and *PtrHB7* may have overlapping functions in regulating vascular development.

The maximum auxin content has been detected in the cambium zone and developing xylem of *Populus* (Schrader *et al*., 2003; Tuominen *et al*., 1997). Meanwhile, the auxin response factor MP/ARF5 has been shown to activate expression of *HD‐ZIP III* genes in vascular tissue of *Populus* (Donner *et al*., 2009; Johnson and Douglas, 2007). Auxin diffusing laterally from the fascicular cambium, which is mediated by polar auxin transport protein PINs, has been suggested to be a key process stimulating formation of the interfascicular cambium (Little *et al*., 2002). *HD‐ZIP III* genes have been shown to affect the expression pattern of *PIN*s during vasculature initiation in leaf, root and lateral growth (Benjamins and Scheres, 2008; Vernoux *et al*., 2010). Expression of *PtrPIN1* and *PtrWOX4*, which was suggested for stimulating cambium activity in an auxin‐dependent manner (Suer *et al*., 2011), was down‐regulated by repression of *PtrHB4* and up‐regulated by overexpression of *PtrHB4*. These results suggest that *PtrHB4* regulation of the interfascicular cambium formation may go through auxin signalling process. However, more detailed characterization is required to elucidate the exact mechanism that *PtrHB4* conducts in interfascicular cambium initiation.

Development of vascular cambium is essential for plant secondary growth which is a critical biological process for sustaining woody plants to grow a long‐life span. Through secondary growth, a large number of photosynthetic products are accumulated in secondary xylem tissue to provide wood, fibre and chemical materials for meeting demands of human society. Finding of the *PtrHB4* role in interfascicular cambium initiation would improve our understanding of the molecular mechanisms underlying vascular cambium formation as well as help develop new strategies to engineer the secondary growth for ideal wood and fibre production.

## Experimental procedures

### Cloning and plant transformation

The full coding sequence of *PtrHB4* and the 2.5 kb promoter of *PtrHB4* of *Populus trichocarpa* were cloned as previous study using the primers in (Table S3) (Zhu *et al*., 2013). *PtrHB4pro:GUS* constructs were generated by replacing the cauliflower mosaic virus 35S promoter of *pBI121* binary vector to the *PtrHB4* promoter. The stop codon of *PtrHB4* was replaced by nucleotide sequence of the SRDX domain using PCR amplification with the reverse primers (Table S3), and then the amplicon of *PtrHB4SRDX* was cloned into *pBI121* vector to generate *35S:PtrHB4SRDX* construct. The overexpression construct *35S:PtrHB4mt* was generated as previous study using one pair of overlapping primers *PtrHB4mt‐F* and *PtrHB4mt‐R* (Table S3). All constructs were transformed into *Populus×euramericana cv. ‘Nanlin895’* by *Agrobacterium*‐mediated transformation according to the protocol adopted in our laboratory (Li *et al*., 2003). For each transformation, at least 25 independent lines of transgenic plants were generated. After transgenic plants were identified and verified by examining transgene expression, the transgenic plants were multiplied through micro‐cutting propagation and used as biological repeats. Among the independent lines which showed morphological phenotype, 2–3 lines with the highest expression of *PtrHB4SRDX* or *PtrHB4mt* were selected for detail characterization (Figures 2c and 5c). All plants in the first 2 months were grown in a phytotron with a light and dark cycle of 16 h and 8 h at 22 °C under a light density of 150 μE/m^2^/s and then moved into a greenhouse with the same light and dark cycle and supplementary light of 200 μE/m^2^/s. Measurement of stem diameter and internode length was taken from the 30th internode from 3‐month‐old plants. Two‐sample *t*‐test was used to determine statistical significance between wild type and individual transgenic lines.

### Gene expression analysis

Total RNA was isolated from tissues of SAM, shoot tip, different stem internodes, and leaves for examining the tissue specificity of *PtrHB4* expression using modified CTAB method (Richards *et al*., 2001). After treatment with DNase I, total RNA was used for first‐strand cDNA synthesis using Hifair™ II 1st Strand cDNA Synthesis SuperMix (Yeasen, http://www.yeasen.com) and followed by RT‐qPCR analysis using gene‐specific primers showing in (Table S3). RT‐qPCR was performed using UNICON™ SYBR Green^®^ Real‐Time PCR Master Mix (Yeasen, http://www.yeasen.com) and an iQ5™ Real‐Time PCR Detection System (Bio‐Rad, http://www.bio-rad.com/) according to the manufacturer's instructions. All reactions were performed with at least three biological repeats and three technical repeats. Statistical analyses (two‐sample *t*‐test) were preformed to evaluated statistical significance between wild type and individual transgenic lines.

### Histochemical and histological analyses

Shoot tips and series of internodes of stem from *PtrHB4pro: GUS* transgenic plants were hand‐sectioned and then incubated in 50% acetone (v/v) for 10 min on ice, and incubated in GUS stain solution (100 mm sodium phosphate (pH7.0), 10 mm EDTA, 0.5 mm ferricyanide, 0.5 mm ferrocyanide, 0.1% Triton X‐100, 20% methanol and 2 mm X‐Gluc) at 37 °C for 2 h. Following staining, sections were cleared by 75% ethanol and photographed. Shoot tips and stem segments with 3 mm length at different internodes from transgenic and wild type plants were fixed in formaldehyde‐acetic acid solution (formaldehyde:glacial acetic acid:ethanol [1 : 1 : 18]) for 24 h, dehydrated in graded ethanol series, and embedded into paraplast. The samples were sectioned to 10 μm thick using rotary microtome of Leica RM2235 (Leica, http://www.leica-microsystems.com/products). The sections were stained with toluidine blue and observed under a light microscope of OLYMPUS BX51 (OLYMPUS). For lignin staining, sections were immersed in 1% phloroglucinol (w/v) in 12% HCl for 5 min and immediately observed with a light microscope.

### Immunolocalization

An N‐terminal‐specific peptide of PtrHB4 (SKDKHMDSSKYVRY) was synthesized and injected into rabbits to raise antibodies (Abmart, Shanghai, China) (Figure S1b). Rabbit serum was collected and purified using protein‐A/G Sepharose. The purified antibodies were diluted into a concentration of 1 μg/μL for later use. The N‐terminal 150 amino acids length of PtrHB4 protein was cloned into pET28 vector and then expressed in *E. coli*. (BL21). Total proteins of cell lysate after IPTG induction were separated by SDS‐PAGE to examination expression of protein (Figure S1c). Western blot was performed using total proteins of cell lysate and from shoot tip of wild type plants against PtrHB4 antibody (diluted at 1 : 1000) to analysis antibody specificity according to the previous protocol (Figure S1d and e) (Song *et al*., 2010). The secondary antibodies (linked with alkaline phosphatase, Santa Cruz, CA) were diluted in 1 : 5000. The shoot tip and internodes of wild plants were embedded and sliced into 10‐μm‐thick sections for immunolocalization according to the previous protocol (Song *et al*., 2010). The first antibodies were diluted in 1 : 200. The secondary antibodies were diluted in 1 : 1000. After colour development, the sections were gradually dehydrated with alcohols, cleared with xylene and observed under an OLYMPUS BX51 light microscope (Olympus, NY).

### RNA sequencing and analysis

Total RNAs were isolated from the shoot tips using modified CTAB method, which were collected from three independent lines of the transgenic and WT plants. The quality of the total RNAs was determined by OD260/OD280 ratios and agarose gel electrophoresis. The concentration of the total RNAs was >400 ng/μL and the total quantity was >20 μg per samples. The RNA sequencing libraries were constructed after combination three biological repeats of transgenic or WT plants as our previous protocol (Zhu *et al*., 2013). The library products were sequenced via Illumina HiSeq™ 2000 with the paired‐end‐100 bp reads. The RNA‐Seq data have been submitted to NCBI Sequence Read Archive (accession number SUB2483520). The raw sequence data were analysed using Illumina HiSeq™ 2000 software. The raw reads were filtered to generate clean reads and then mapped to the *Populus trichocarpa* genome using SOAPalibner/soap2 (Li *et al*., 2009). The reads with no more than two bases of mismatch were used for alignment. Gene annotation was on the basis of the *Arabidopsis* genome database (TAIR10). The transcript level was calculated using the RPKM (Reads Per kb per Million reads) method (Mortazavi *et al*., 2008). The expression difference between *PtrHB4SRDX* and WT was examined with the threshold of P‐value in multiple tests (Audic and Claverie, 1997; Benjamini *et al*., 2001). A total of 45 786 334 and 47 587 472 cDNA reads were identified from samples of *PtrHB4SRDX* and WT plants. 81.27% and 81.42% of these reads were mapped to the *Populus trichocarpa* genome and 47 445 and 47 703 genes were detected in the two samples, respectively. GO enrichment analysis was performed as described in (Xue *et al*., 2013).

## Supporting information


**Figure S1** Expression of *PtrHB4* during vascular cambium development.

**Figure S2** Interfascicular cambium development in stems of *Populus*.

**Figure S3** Construct of *35S:PtrHB4SRDX*.

**Figure S4** Repression of *PtrHB4* affected xylem development.

**Figure S5** Mutations in the miRNA166 target sites.

**Figure S6** Overexpression of *PtrHB4mt* transformed vascular bundles pattern to amphivasal in *Arabidopsis*.

**Table S1**List of the down‐regulated genes in *PtrHB4SRDX* plants.

**Table S2** List of the up‐regulated genes in *PtrHB4SRDX* plants.

**Table S3** List of primers used in this study.
