# Supplementary figures and images for: A HD‐ZIP III gene, PtrHB4, is required for interfascicular cambium development in Populus
Source: Plant Biotechnol J. 2017 Nov 18;16(3):808–17. doi: 10.1111/pbi.12830 (PMC5814583; doi:10.1111/pbi.12830)

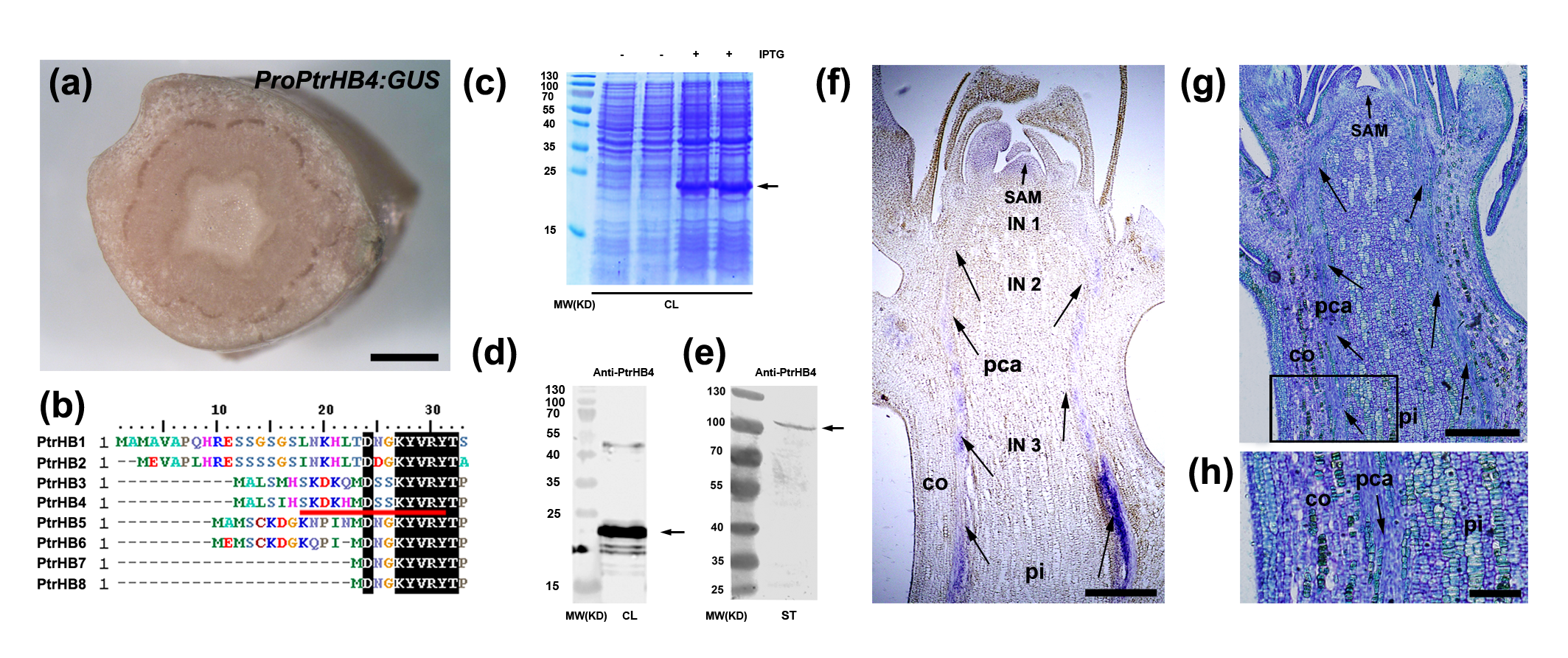

Supplement: Supplementary file 1 — Figure S1 Expression of PtrHB4 during vascular cambium development. [file PBI-16-808-s004.tif]

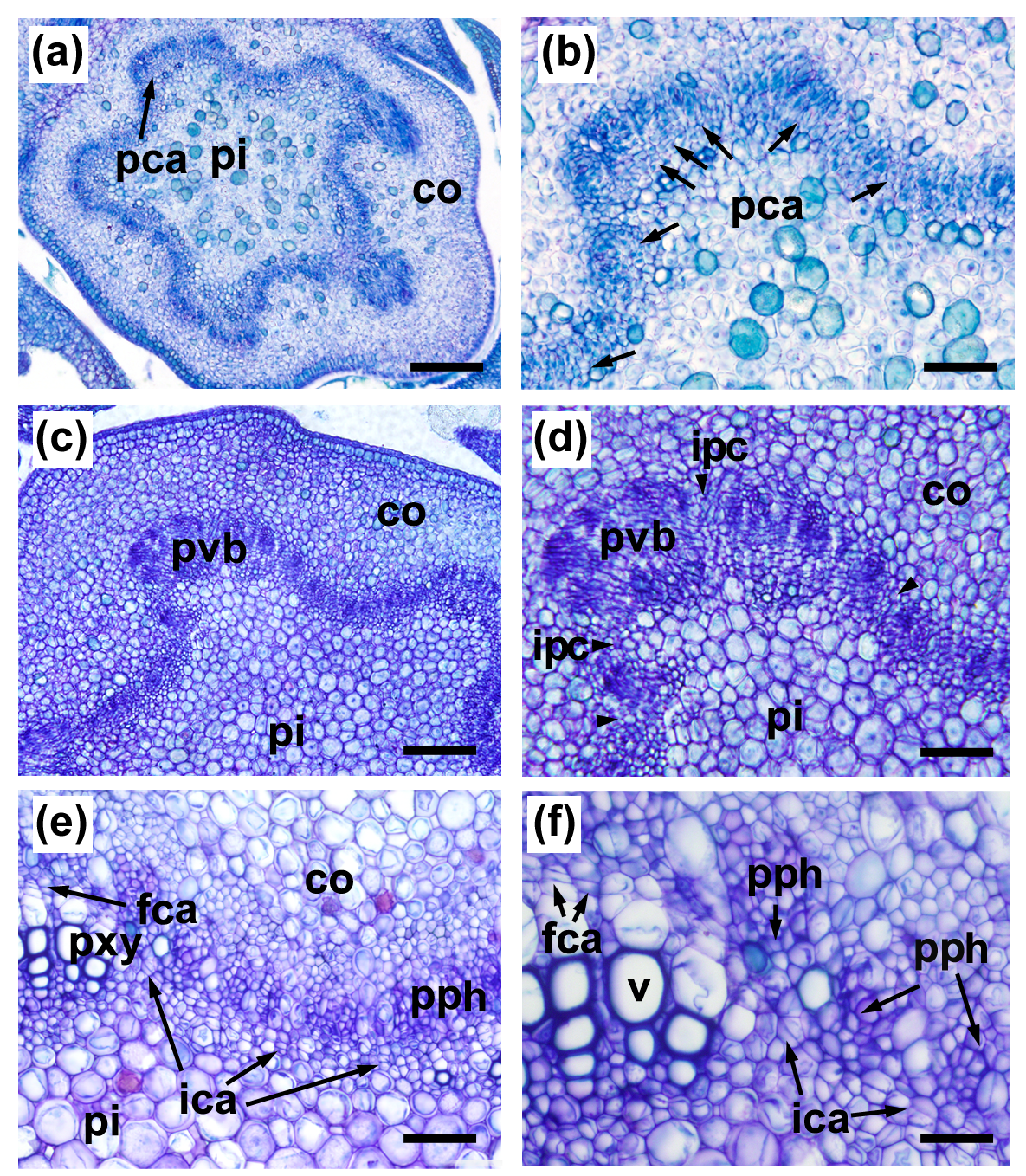

Supplement: Supplementary file 2 — Figure S2 Interfascicular cambium development in stems of Populus. [file PBI-16-808-s007.tif]

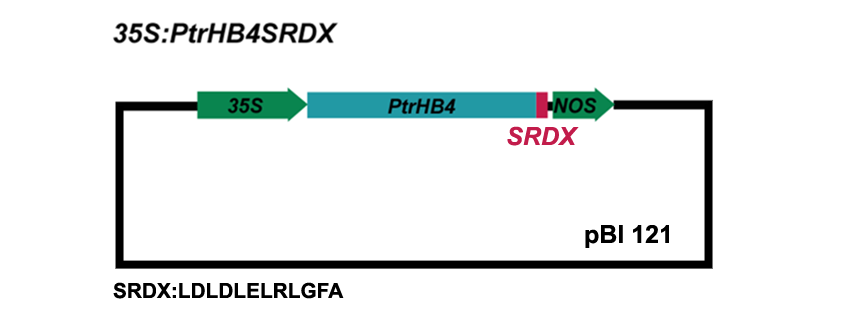

Supplement: Supplementary file 3 — Figure S3 Construct of 35S:PtrHB4SRDX. [file PBI-16-808-s006.tif]

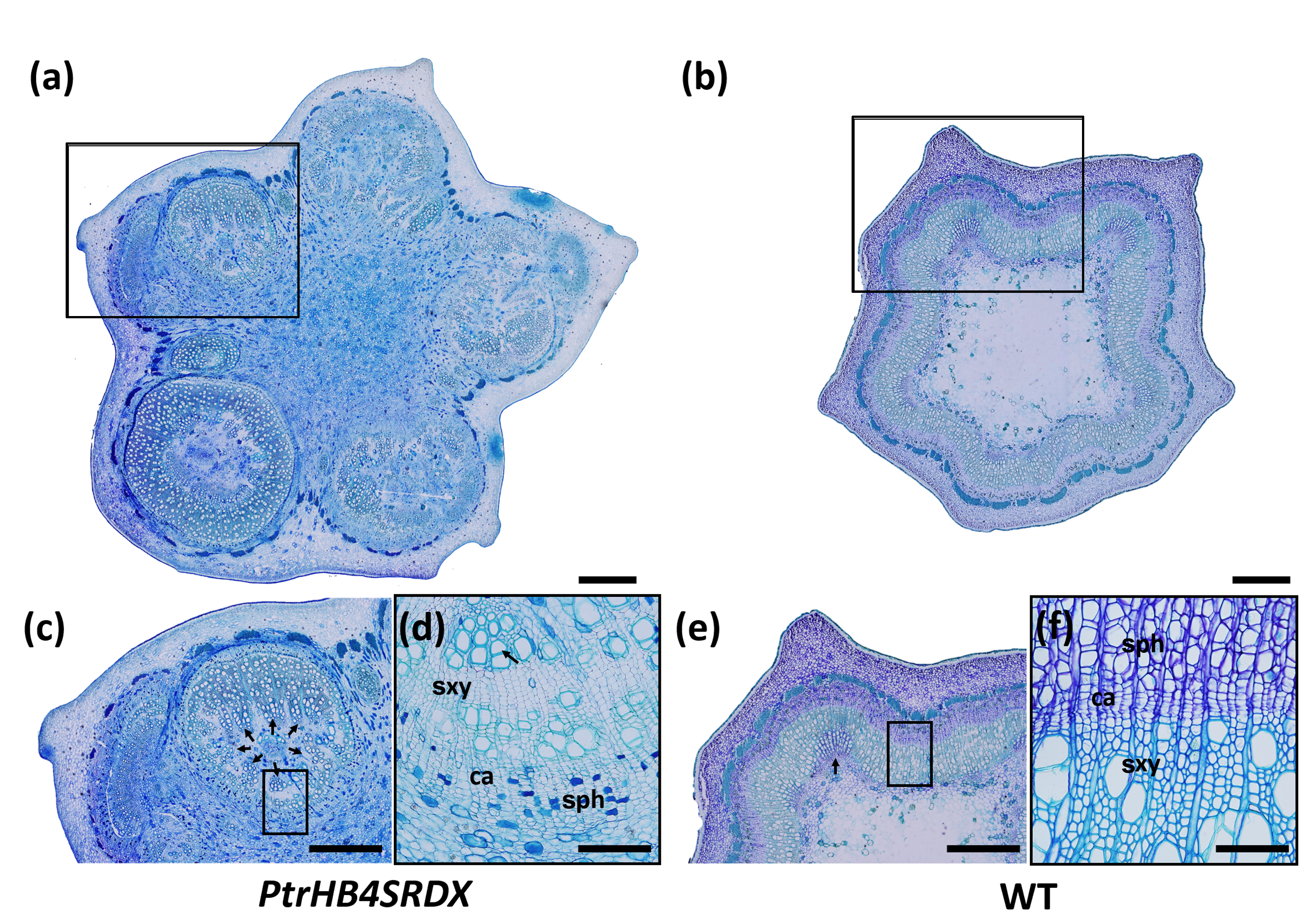

Supplement: Supplementary file 4 — Figure S4 Repression of PtrHB4 affected xylem development. [file PBI-16-808-s008.tif]

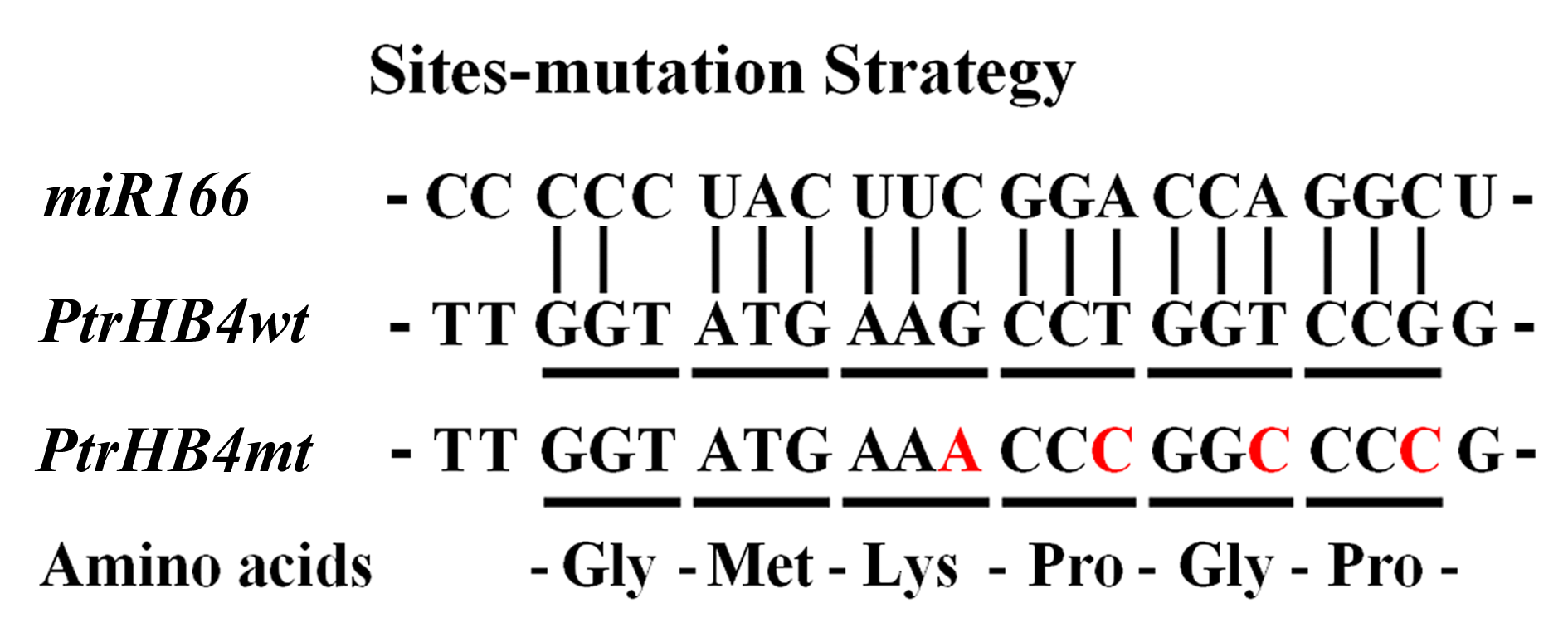

Supplement: Supplementary file 5 — Figure S5 Mutations in the miRNA166 target sites. [file PBI-16-808-s001.tif]

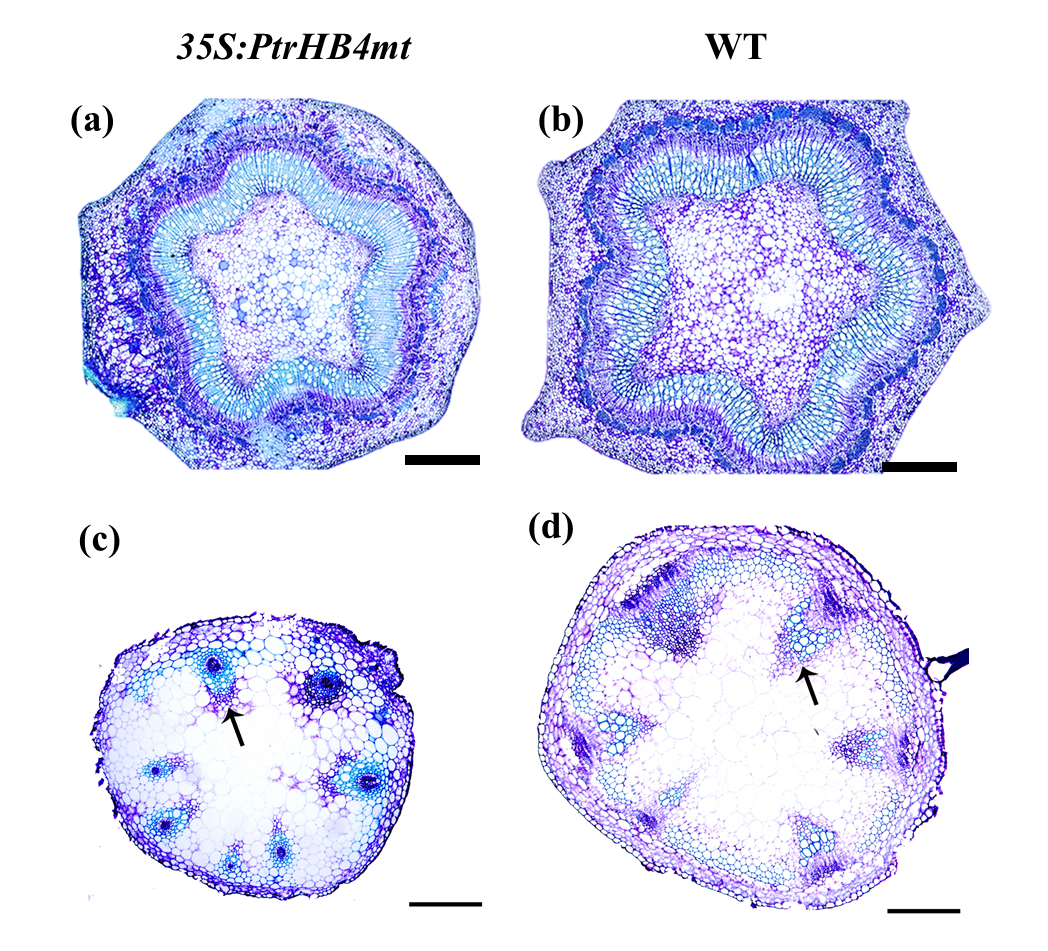

Supplement: Supplementary file 6 — Figure S6 Overexpression of PtrHB4mt transformed vascular bundles pattern to amphivasal in Arabidopsis. [file PBI-16-808-s002.tif]
